# Aggressive Neoplasms That Mimic Chronic Endodontic Lesions: A Multi‐Institutional Case Series

**DOI:** 10.1111/aej.12956

**Published:** 2025-05-30

**Authors:** Natália Gonçalves Macedo, Fernanda Geraldo Pappen, Bernardo da Fonseca Orcina, Gustavo Mascarenhas, Jean Nunes dos Santos, Paulo Sérgio da Silva Santos, Carolina Clasen Vieira, Alexandre Simões Garcia, Alini Cardoso Soares, Lauren Frenzel Schuch, Ana Paula Neutzling Gomes, Ana Carolina Uchoa Vasconcelos

**Affiliations:** ^1^ Diagnostic Center for Oral Diseases, School of Dentistry Federal University of Pelotas Pelotas Rio Grande do Sul Brazil; ^2^ Department of Clinical Semiology Federal University of Pelotas Pelotas Rio Grande do Sul Brazil; ^3^ Private Practitioner Salvador Bahia Brazil; ^4^ Department of Oral and Maxillofacial Pathology, School of Dentistry Federal University of Bahia Salvador Bahia Brazil; ^5^ Department of Surgery, Stomatology Pathology and Radiology—Bauru School of Dentistry of University of São Paulo Bauru São Paulo Brazil; ^6^ Molecular Pathology Area, School of Dentistry Universidad de la República—UDELAR Montevideo Uruguay

**Keywords:** diagnostic errors, head and neck neoplasms, neoplasms, non‐endodontic lesion, periapical diseases

## Abstract

This study aimed to report sociodemographic, clinicopathologic and radiological features of 12 cases of aggressive neoplasms that were clinically diagnosed as chronic endodontic lesions. The series comprised seven females (58.33%) and five males (41.67%), with a mean age of 48.9 ± 19.19 years (range: 16–75 years). In six cases (50%), the posterior maxillary region was involved. Internal appearance was radiolucent density (*n* = 12/100%). Eleven cases (91.67%) exhibited well‐defined borders, while one (8.33%) showed ill‐defined borders. Histopathologic diagnoses included metastasis (*n* = 4/33.33%), squamous cell carcinoma (*n* = 3/25%), mucoepidermoid carcinoma (*n* = 2/16.68%), acinic cell carcinoma (*n* = 1/8.33%), Ewing's sarcoma (*n* = 1/8.33%) and Langerhans cell histiocytosis (*n* = 1/8.33%). Survival status was retrieved for three patients (25.00%), all of whom were alive at the time of reporting. This study identified seven distinct histological types of aggressive neoplasms that mimic chronic periapical lesions. Given that such lesions often lack clinical or radiological signs of aggressive behaviour, it is imperative that all excised tissue undergo histopathological examination.

## 1 Introduction

Periapical lesions are typically sequelae of pulpal necrosis and arise from either bacterial infection or trauma. These endodontic lesions may be chronic (periapical cyst, periapical granuloma or chronic abscess) or acute (periradicular abscess or cellulitis), and they represent the commonest radiolucencies in the jaws [1, 2, 3]. It is estimated that approximately 90% of radiolucent or hypodense jawbone pathologies with a presumptive radiographic diagnosis of chronic endodontic lesions are either periapical cysts or periapical granulomas [4, 5].

Several studies have demonstrated that a range of benign and malignant pathologies can clinically and radiographically mimic sequelae of pulpal necrosis (SPN), such as periapical cysts and granulomas [6]. Approximately 4% of the lesions with a clinical and radiographic diagnosis of chronic periapical disease, when submitted to histopathological examination, reveal conditions that do not arise from a pulp pathology [6]; of these, it is estimated that 0.39% to 3.7% are malignant neoplasms [4, 7]. Metastatic neoplasms, malignant salivary gland neoplasms and lymphomas comprise some aggressive non‐SPN reported in previous studies [4, 8]. A recent systematic review of periapical malignant non‐endodontic neoplasms has shown that these conditions have a slight female predilection and a higher incidence in individuals in the sixth decade of life [8]. The same authors reported that the posterior region of the mandible is the most commonly affected and they present mostly as periapical radiolucencies [8].

Misdiagnosis of aggressive non‐SPN lesions (ANSPNLs) can lead to delays in initiating antineoplastic treatment, which directly impacts patient survival [8]. Thus far, few cases of ANSPNLs with enough clinicopathologic and imaginological details have been documented in the English‐language literature [9, 10]. Case series on ANSPNLs remain rare; therefore, consistent documentation is essential to support a better understanding of these lesions. This study aimed to present the sociodemographic, clinicopathologic and radiological features of 12 cases of aggressive neoplasms with a presumptive diagnosis as chronic periapical lesions.

## 2 Material and Methods

### 2.1 Ethical Issues, Sample and Study Design

In the present series, a convenience sample comprising 12 ANSPNL cases was retrieved from the archives of three Oral and Maxillofacial Pathology services: the Department of Oral Pathology of the Federal University of Bahia (Northeast region), the Department of Oral Pathology and Surgery of the University of São Paulo (Southeast region) and the Diagnostic Center for Oral Diseases of the Federal University of Pelotas (South region). The cases were diagnosed between December 1999 and February 2023. The study was approved by the Institutional Ethics Committee (No. 2.887.524) and was conducted in accordance with the Declaration of Helsinki and Strengthening the Reporting of Observational Studies in Epidemiology (STROBE) guidelines [11]. Informed consent was obtained from all individual participants included in the study.

### 2.2 Data Collection

Presumptive diagnoses were made by clinicians, endodontists and oral and maxillofacial surgeons. Inclusion criteria were based on the clinical and radiographic diagnostic features consistent with chronic endodontic lesions (periapical granuloma, periapical cyst and chronic abscess). Accordingly, cases lacking periapical radiographs, orthopantomograms or computed tomography scans were excluded.

Histopathological data were independently reviewed by three oral and maxillofacial pathology specialists (J.N.S., A.P.N.G. and A.S.G.), each with over 20 years of experience. Any disagreements among the evaluators were resolved through discussion and consensus. While assessments primarily relied on existing materials, in some instances—particularly when older records were involved—new slides were prepared for evaluation. All histopathological diagnoses were rendered in accordance with the most recent World Health Organization (WHO) classification [12].

When available, the following data were collected: patient sex, age, symptoms, disease duration (months), radiographic features (information about internal appearance, image definition [well/ill‐defined], effects of the lesion on surrounding structures [cortical bone]), anatomical location (jaws were classified into anterior—lesions in the incisor/canine region—and posterior—lesions in the premolar/molar retromolar/ramus/maxillary sinus region), histopathological diagnosis, follow‐up (months) and status (death/alive).

### 2.3 Statistical Analysis

Descriptive statistical analyses of demographic data and clinical‐radiological features were performed using the Statistical Package for the Social Sciences software (SPSS; IBM Corp., version 23.0, Armonk, US).

## 3 Results

**FIGURE 1:**
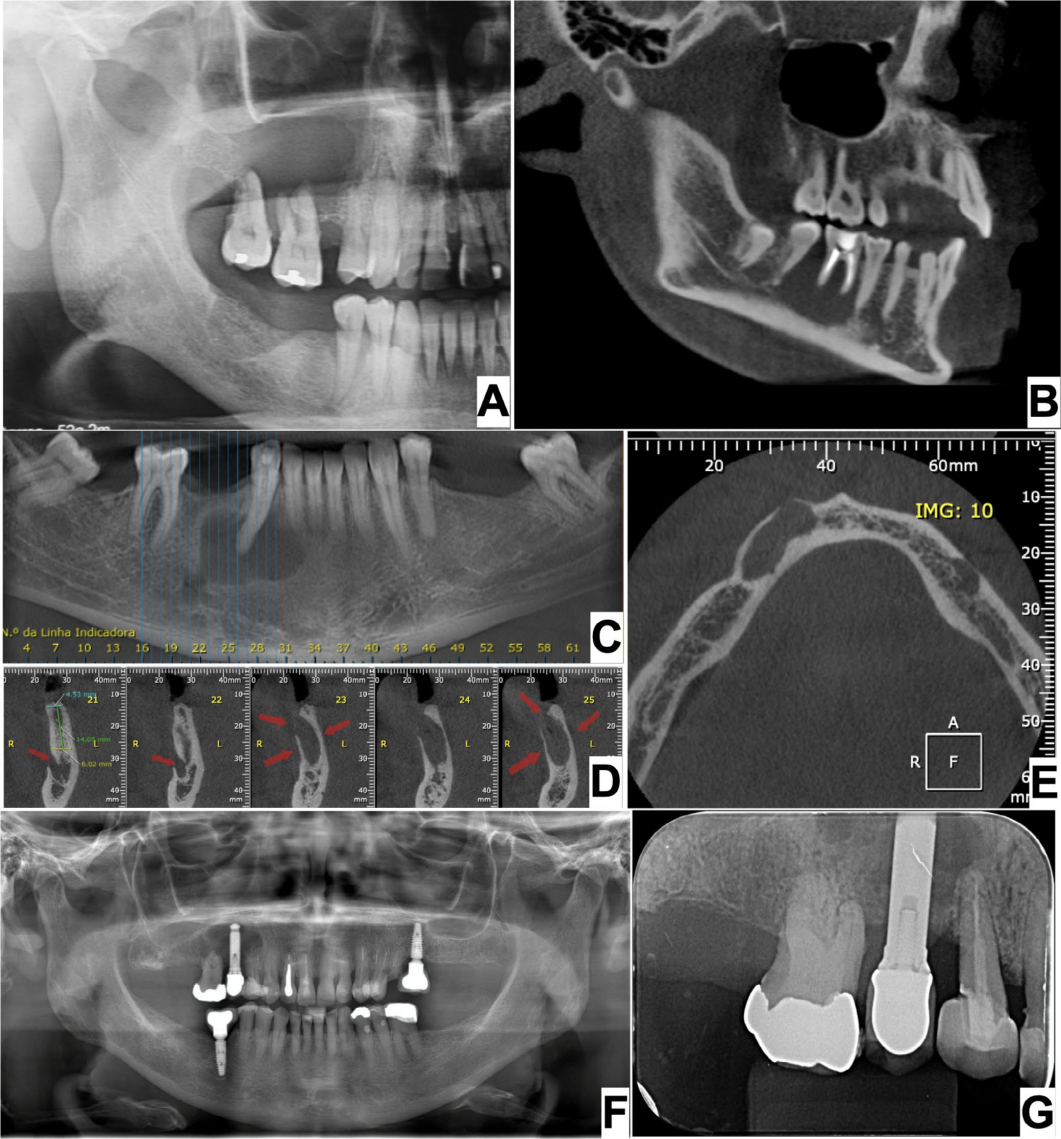
Radiographic features of ANSPNL of some included cases. Metastasis: (A) A panoramic radiograph showing radiolucent, unilocular and well‐defined lesion in the upper right molar region. Ewing sarcoma: (B) A tomography showing a hypodense, unilocular and well‐defined lesion in lower left molar region. Squamous cell carcinoma (SCC): (C) Cone beam computed tomography panoramic view revealing a hypodense, unilocular lesion with a well‐defined/punched‐out margin between the right lateral incisive and premolar. (D) Sagittal view. The red arrows in the images indicate the sites of cortical bone perforation. (E) Coronal view revealing a hypodense lesion with no perforation of buccal cortical bone. Langerhans cell Histiocytosis (LCH): (F) Panoramic radiograph showing a radiolucent lesion in the upper right premolar region. (G) A periapical radiograph of the same patient reveals a radiolucent, unilocular and well‐defined lesion.

The demographic, clinical and radiographic characteristics of the sample are presented in Table 1. Seven cases (58.33%) occurred in females and five (41.67%) in males (female to male ratio 1.4:1). The mean age at diagnosis was 48.9 ± 19.19 years (range: 16–75 years). The mean duration of disease was 15.33 ± 14.55 months (range: 2–36 months) and symptoms were reported in four patients (33.34%) of five informed cases. With respect to anatomical location, six cases (50%) were located in the posterior maxilla, three (25.00%) in the posterior mandible, two (16.67%) in the anterior mandible and one (8.33%) in the anterior maxilla. Radiographic analysis revealed 11 cases (91.67%) with well‐defined borders and one (8.33%) with ill‐defined borders. The internal appearance was predominately radiolucent/radiodense (Figure 1A,C,D). Cortical bone perforation was identified in two cases (16.67%) and expansion of the cortical bone was detected in one case (8.33%) (Figure 1G).

**TABLE 1:** Demographic, clinical and radiographic characteristics of the sample.

Variable	*n* (%)
Sex (*n* = 12)	
Female	7 (58.33)
Male	5 (41.67)
Male‐to‐female	1.4:1
Age (*n* = 12)	Mean: 48.9 ± 19.19 years
	Range:16–75 years
Diseases duration (*n* = 6)	Mean: 15.33 ± 14.55 years
	Range: 2–36 years
Symptoms (*n* = 12)	
Pain	4 (33.34)
Asymptomatic	1 (8.33)
Not informed	7 (58.33)
Anatomical location (*n* = 12)	
Anterior maxilla	1 (8.33)
Posterior maxilla	6 (50.00)
Anterior mandible	2 (16.67)
Posterior mandible	3 (25.00)
Radiographic features (*n* = 12)	
Internal appearance (*n* = 12)	
Radiolucent	12 (100.00)
Image definition (*n* = 12)	
Well‐defined	11 (91.67)
Ill‐defined	1 (8.33)
Cortical bone perforation (*n* = 12)	
Yes	2 (16.67)
No	10 (83.33)
Expansion of the cortical bone (*n* = 12)	
Yes	1 (8.33)
No	11 (91.67)
Histopathological diagnosis (*n* = 12)	
Metastasis (*n* = 4)	
Adenocarcinoma metastasis	2 (16.67)
Kidney metastasis	2 (16.67)
Malignant neoplasm of salivary gland (*n* = 3)	
Mucoepidermoid carcinoma	2 (16.67)
Acinic cell carcinoma	1 (8.33)
Squamous cell carcinoma	3 (25.00)
Ewing's sarcoma	1 (8.33)
Langerhans cell histiocytosis	1 (8.33)
Follow‐up (*n* = 3)	Mean: 45.3 ± 44.6 months
	Range: 12–96 months
Status/Treatment (*n* = 12)	
Alive	3 (25.00)
Surgical treatment	3 (100.00)
Not informed	9 (75.00)

**FIGURE 2:**
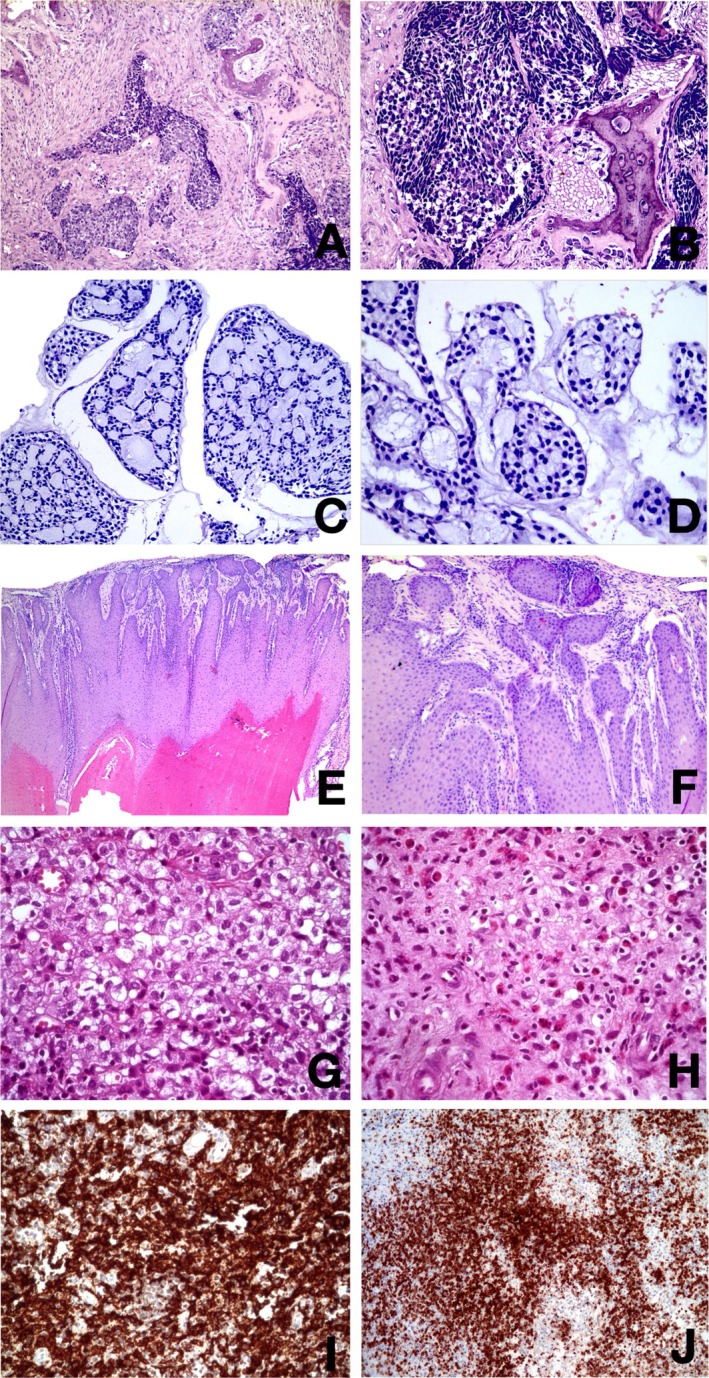
Representative photomicrographs of ANSPNLs. Microscopic aspects of metastasis: (A, B) Infiltration by a solid tumour arranged in cords and nests. Individual tumour cells are round to polygonal with well‐defined borders, clear to eosinophilic cytoplasm and ovoid hyperchromatic nuclei (Haematoxylin and eosin (H&E) stain, 100× and 200×, respectively). Histologic picture showing the arrangement of Mucoepidermoid carcinoma (MEC): (C, D) prominent cribriform patterns of growth. The cribriform areas show pseudocysts and hyalinized material (H&E stain, 200× and 400×, respectively). Microscopic aspects of squamous cell carcinoma: (E, F) Infiltration characterised by blunt and elongated projections, forming nests and islands within a solid tumour arranged in cords and nests. Invasion of nests and islands of dysplastic epithelium into the connective tissue (H&E stain, 40× and 100×, respectively). Microscopic aspects of Langerhans cell histiocytosis (LCH): (G) LCH showing a clear cytoplasm and nucleus with indentations similar to coffee beans or kidney‐shaped (Haematoxylin and eosin [H&E] stain, 200×). (H) Dense inflammatory infiltrate with the presence of eosinophils (H&E stain, 200×). Immunohistochemical profile of the same patient: (I) CD1a‐positive immunostaining (original magnification 200×) and (J) CD5‐positive immunostaining (original magnification 100×).

Figure 2 illustrates selected histopathologic diagnoses. Metastatic lesions were the most common group (*n* = 4/33.33%) (Figure 2A,B). Malignant neoplasms of the salivary glands (MNSG) were the second most frequent lesions (*n* = 3/25%), comprising two cases (16.68%) of mucoepidermoid carcinoma (MEC) and one case (8.33%) of acinic cell carcinoma (ACC) (Figure 2G,H). Squamous cell carcinomas (SCC) (*n* = 3/25%) were the third most common ANSPNL. Ewing's sarcoma (ES) (*n* = 1/8.33%) and Langerhans cell histiocytosis (LCH) (*n* = 1/8.33%) (Figure 2C–F) were also identified. Follow‐up was available for two cases, with a mean time of 45.3 ± 44.6 months. Survival status was retrieved for three (25.00%) cases, and all patients were alive at the time of reporting (Table S1).

## 4 Discussion

Malignant lesions of the oral cavity account for approximately 5%–6% of all cancers affecting the human body [13]. To date, only a few well‐documented cases of ANSPNL of the jaws have been reported in the literature, highlighting the rarity of this condition [5]. In a study of non‐endodontic periapical lesions misdiagnosed as endodontic periapical pathoses obtained from biopsies of the apical region, Huang et al. (2017) observed that only nine of the 118 lesions, that is, 7.62% of the sample, were categorised as malignant neoplasms [14]. Similarly, Vieira et al. (2020) detected 10 malignant cases among 306 cases of misdiagnosed endodontic lesions [4]. Overall, seven different histopathological diagnoses were observed in the present case series.

In the present study, metastatic lesions represented the most frequent group. Metastases are rare in the oral regions, accounting for approximately 1% of all malignant diseases in this area [5, 13]. In a recent systematic review, Schuch et al. (2021) analysed 60 malignant lesions that mimicked chronic endodontic lesions, with metastatic jaw lesions being the most frequently reported, similar to our findings [8]. The authors also reported a marked predilection for the mandible—particularly in the posterior region, which was similarly observed in the present series [8]. Kirschnick et al. (2022) evaluated 348 cases of metastatic lesions in the oral and maxillofacial region and observed that males were more commonly affected, primarily in the 5th to 7th decades of life. Their findings also indicated a preference for bone tissue—especially the posterior mandible—when compared to soft tissue sites (ratio of 1.3:1) [15]. In the four metastatic cases reported in the present survey, no sex preference was noted, and individuals were most commonly affected in their sixth decade of life. The development of jawbone metastases is a complex process involving crosstalk between disseminated cells and bone‐derived molecules. This leads to deregulating signalling pathways critical for normal bone remodelling processes [15]. The posterior region of the mandible seems to have remnants of active haematopoietic marrow, especially in osteoporotic defects, which can explain the preference for metastatic cell growth in this region [15]. Although jaw metastasis does not involve a pathognomonic clinical‐radiographic presentation, symptoms such as pain, paresthesia and lytic radiolucent lesions with ill‐defined margins are frequently detected [15]. Curiously, all four metastatic cases presented as well‐defined radiolucent lesions, with cortical bone perforation observed in only one of them. Unfortunately, the prognosis for patients with jawbone metastases remains poor, with reported overall survival rates of approximately 17.7% at 3 years and 7.3% at 5 years [15].

In the present study, MNSG accounted for three cases, representing the second most recurrent group in the sample. Ectopic salivary gland tissue has been identified not only within jawbones but also in diverse anatomical sites, including the nasal skin and cervical lymph nodes [14]. In addition, neoplastic transformation of the sinus epithelium and the epithelial lining of an odontogenic cyst has been proposed as a potential origin of central malignant tumours of the jaws [16]. In a literature review, Li et al. (2008) summarised the clinical and demographic data of 197 cases of central jaw MNSG, found that MEC, followed by adenoid cystic carcinoma, was the most common histopathological diagnosis [9]. This partially aligns with our findings, which include two cases of MEC and one case of ACC. An exceedingly rare variant of MEC is known as intraosseous MEC, occurs more commonly in the mandible than the maxilla and exhibits a slight male predilection. Interestingly, both MEC cases in the present series were located in the posterior maxilla. Although those lesions present predominantly well‐defined radiographic appearance, it is difficult to discriminate whether the tumour in the maxilla perforated the cortical plates or the tumour of enclosing tissues (palate, gingival mucosa of maxillary sinus) invaded the bony tissue. Unfortunately, data about the presence or absence of any primary tumour in minor salivary glands were not obtained. Moreover, computed tomographic imaging was not performed, making it difficult or impossible to ascertain the true origin of the lesions.

The present study shows that SCC represents 25% of the sample. Primary intraosseous squamous cell carcinoma (PIOSCC) is defined as a carcinoma arising within the jaw, with no initial connection with the oral mucosa and presumably developing from the residue of odontogenic epithelium or odontogenic cysts or tumour [13]. The underlying mechanisms triggering proliferation of odontogenic epithelial remnants remain poorly understood; however, chronic inflammatory stimuli–potentially in combination with genetic predisposition–are considered likely contributors to neoplastic transformation [17]. Huang et al. (2009) published a review of 39 cases of PIOSCC which showed that the lesion affects patients aged 24 to 82 years, with a mean age at diagnosis of 54 years. The male‐to‐female ratio was 2:1 and the tumours occurred predominantly in the posterior mandible [18]. These findings are partially consistent with the three cases of SCC presented, all of which occurred in men with a mean age of 53.66 years. Curiously, only one of the three cases was located in the posterior mandible. Due to the nature of this retrospective report, the diagnosis of PIOSCC is challenging, mainly because it is difficult to distinguish such lesions from alveolar carcinomas invading bone from adjacent soft tissue. In addition, the prognosis for patients with PIOSCC is challenging to determine because of the small number of reported cases, the different treatment modalities and the variable follow‐up time. The overall survival rate of patients with PIOSCC was 40%–68% at 2 years [19]. Notably, one SCC patient in the present study remains alive after 28 months of follow‐up.

ES and LCH are rare neoplasms that mainly affect the paediatric population with unusual involvement of the jaws [20]. Margaix‐Muñoz et al. (2017) analysed 71 cases of ES located in the oral and maxillofacial region. The lesion showed a slight female predominance, with a mean patient age of 15 years, and was primarily located in the mandible presenting as symptomatic. Interestingly, they demonstrated that one out of every five cases was initially mistaken for dental infection [21]. Taken together, all of this clinical and demographic data aligns with the present ES case. It is widely recognised that jaw sarcomas exhibit distinct biological behaviour compared to those in anatomical locations, typically being smaller and less often associated with distant metastasis [22].

LCH is currently defined as a myeloid dendritic cell neoplasm [23, 24]. Its exact incidence in adults remains unclear, with estimates ranging from 1 to 1.5 cases per million people per year, underscoring the rarity of the current case [25]. The classic radiographic appearance of ‘floating teeth’ and preference for the mandible are common primary findings that were not observed in our LCH case [23]. In a retrospective study of 45 cases of LCH with primary manifestation in the orofacial region [23], Capodiferro et al. (2020) reported an overall mortality rate of < 9%. The patients with ES and LCH described here underwent surgical treatment and demonstrated favourable outcomes, with follow‐up durations of 8 years and 12 months, respectively.

It's important to acknowledge certain limitations. First, the study employed a retrospective design, which means that some cases originated in the 1990s, rendering data retrieval unfeasible. Similarly, critical information such as the primary tumour location in cases of metastasis could not be recovered. Also, although most cases originated from dental schools, it is not possible to confirm the reliability of the clinical‐imaginological diagnoses provided by the submitting clinicians. Furthermore, for most of the cases, follow‐up and/or treatment data were unavailable, as treatment was conducted outside the dental school setting (i.e., by medical specialists), and such updates were not systematically recorded. Nonetheless, despite the typical limitations associated with retrospective investigations, we believe that our sample offers valuable clinical and demographic insights into ANSPNLs, which are challenging to study in larger populations.

In summary, ANSPNLs represent a heterogeneous group of lesions with diverse management approaches and prognosis. The lesions most commonly affect individuals in their 5th decade of life. Among the 12 cases examined, seven distinct histological types were identified, including metastasis, SCC and MEC. These findings highlight the critical need for clinicians to remain vigilant to the wide range of malignancies that may clinically and radiographically mimic periapical cysts and granulomas.

## Author Contributions

Conception and design: Natália Gonçalves Macedo and Ana Carolina Uchoa Vasconcelos. Material preparation and data collection: Natália Gonçalves Macedo, Bernardo da Fonseca Orcina, Gustavo Mascarenhas, Jean Nunes dos Santos, Paulo Sérgio da Silva Santos, Carolina Clasen Vieira, Alexandre Simões Garcia, Lauren Frenzel Schuch, Ana Paula Neutzling Gomes and Ana Carolina Uchoa Vasconcelos. Analysis and interpretation of data collection: Natália Gonçalves Macedo, Alini Cardoso Soares and Ana Carolina Uchoa Vasconcelos. Drafting of the manuscript and critical revision: Fernanda Geraldo Pappen, Jean Nunes dos Santos and Ana Carolina Uchoa Vasconcelos. All authors read and approved the final manuscript.

## Ethics Statement

In the present series, cases of ANELs of the jaws were retrieved from the archives of three Brazilian Oral and Maxillofacial Pathology services: the Department of Oral Pathology of the Federal University of Bahia (Northeast region), the Department of Oral Pathology and Surgery of the University of São Paulo (Southeast region), and the Diagnostic Center for Oral Diseases of the Federal University of Pelotas (South region). The study was approved by the Institutional Ethics Committee (No. 2.887.524) and was conducted in agreement with the Declaration of Helsinki.

## Conflicts of Interest

The authors declare no conflicts of interest.

## Supporting information


Table S1.

## Data Availability

The data that support the findings of this study are available from the corresponding author upon reasonable request.
