# Supplementary material for: Aggressive Neoplasms That Mimic Chronic Endodontic Lesions: A Multi‐Institutional Case Series
Source: Aust Endod J. 2025 May 30;51(2):415–22. doi: 10.1111/aej.12956 (PMC12351104; doi:10.1111/aej.12956)
Supplement: Supplementary file 1 — Table S1. [file AEJ-51-415-s001.docx]

**Supplementary Table 1.** Demographic and clinical data, radiographic features and histopathological diagnosis of individuals

| **Case** | **Sex/Age**  **(years)** | **Symptoms** | **Evolution time (months)** | **Internal**  **appearance** | **Image**  **definition** | **Cortical bone**  **perforation** | **Anatomical location** | **Histopathological diagnosis** | **Follow-up (months)** | **Status** |
| --- | --- | --- | --- | --- | --- | --- | --- | --- | --- | --- |
| 01 | F/51 | NI | NI | Radiolucent | Well-defined | NI | Posterior mandible | Adenocarcinoma metastasis | NI | NI |
| 02 | M/45 | NI | 3 | Radiolucent | Well-defined | NI | Posterior maxilla | Squamous cell carcinoma | NI | NI |
| 03 | M/71 | NI | NI | Radiolucent | Well-defined | NI | Anterior maxilla | Squamous cell carcinoma | NI | NI |
| 04 | F/26 | NI | 24 | Radiolucent | Well-defined | NI | Posterior maxilla | Mucoepidermoid carcinoma | NI | NI |
| 05 | F/65 | NI | 3 | Radiolucent | Well-defined | NI | Posterior maxilla | Acinic cell carcinoma | NI | NI |
| 06 | M/42 | Yes | NI | Radiolucent | Well-defined | Yes | Anterior mandible | Adenocarcinoma metastasis | NI | NI |
| 07 | F/28 | NI | NI | Radiolucent | Well-defined | NI | Posterior maxilla | Mucoepidermoid carcinoma | NI | NI |
| 08 | M/52 | NI | NI | Radiolucent | Well-defined | NI | Posterior maxilla | Kidney metastasis | NI | NI |
| 09 | F/75 | Yes | 24 | Radiolucent | Well-defined | NI | Posterior mandible | Kidney metastasis | NI | NI |
| 10 | M/45 | No | NI | Radiolucent | Well-defined | NI | Anterior mandible | Squamous cell carcinoma | 28 | Alive |
| 11 | F/16 | Yes | 2 | Radiolucent | Ill-defined | Yes | Posterior mandible | Ewing’s sarcoma | 96 | Alive |
| 12 | F/71 | Yes | 36 | Radiolucent | Well-defined | NI | Posterior maxilla | Langerhans cell histiocytosis | 12 | Alive |

*F, female; M, male; NI, Not informed.
